# Clinical Informatics Education to Advance Learning Health Systems: A Scoping Review

**DOI:** 10.1002/lrh2.70050

**Published:** 2025-12-05

**Authors:** Alexandra Zingg, L. Ida Tovar, Laura Witte, Kelsey L. Koym, Kyler Godwin

**Affiliations:** ^1^ Health Professions Education, Evaluation, and Research (HPEER) Fellowship. Center for Innovations in Quality, Effectiveness, and Safety Houston Texas USA; ^2^ Health Professions Education, Evaluation, and Research (HPEER) Fellowship. Salt Lake City VA Healthcare System Salt Lake City Utah USA; ^3^ U.S. Department of Veterans Affairs Houston Texas USA; ^4^ Texas Medical Center Library Houston Texas USA; ^5^ Implementation Science and Innovation Program. Center for Innovations in Quality Effectiveness, and Safety Houston Texas USA; ^6^ Department of Medicine Baylor College of Medicine Houston Texas USA

**Keywords:** clinical informatics, curriculum design, health professions education, medical students, postgraduate medical professionals

## Abstract

**Introduction:**

Learning health systems leverage clinical data and knowledge to advance healthcare quality. Effective training in informatics concepts and tools is essential for medical trainees to become health system experts and contributors to positive organizational change. The objective of this study is to summarize characteristics of existing clinical informatics training programs and map these to recommended Learning Health System informatics competencies. We aim to answer the following research questions: (1) How are academic medical institutions implementing informatics education initiatives for medical trainees? (2) Are these initiatives implementing recommended informatics competencies? and (3) How effective are these initiatives according to established health professions education evaluation frameworks?

**Methods:**

We searched for literature in the databases Embase, Ovid Medline, and Web of Science. Three researchers independently screened study titles and abstracts. Inclusion criteria were (a) studies with medical students and/or postgraduate medical professionals in the study sample, (b) in an academic medical setting, and (c) describing a clinical informatics curriculum initiative.

**Results:**

We included a total of 27 studies for analysis. Most curricula (*n* = 16) had the objective of basic informatics knowledge acquisition. Instruction delivery for most (*n* = 17) included a combination of didactic and practical components. The most common evaluation tool was student self‐reported confidence and self‐efficacy. All but three of the studies integrated the recommended informatics competency of demonstrating knowledge of clinical information systems such as Electronic Health Records. Most studies (*n* = 12) reported outcomes related to the Kirkpatrick Level II of Learning.

**Conclusion:**

Gaps remain in the context of leveraging education to pursue Learning Health System endeavors of clinical research, quality improvement, and achieving organizational‐level results. Further research on recent education initiatives targeting undergraduate and graduate medical trainees is needed to elevate the rate of clinical informatics education implementation, while simultaneously advocating for standardization in the design and evaluation of these initiatives.

## 1 Introduction

In learning health systems (LHS), health data and experience are combined with existing evidence, and the resulting knowledge is turned into practice [1]. LHS can advance healthcare quality through mitigating risk for errors and missed opportunities for care, examples being disease risk prediction and improving diagnosis accuracy [2]. Importantly, they can also reduce healthcare costs and financial burden [3]. A crucial factor for LHS to flourish and achieve sustainability is the training of clinicians as LHS research scientists [4]. The Agency for Healthcare Research and Quality (AHRQ) has defined seven competency domains to guide the design and development of LHS training curricula for healthcare professionals [5]. One of these competencies is informatics, which plays a cornerstone role in the genesis of information tools (e.g., electronic health records, clinical decision support, and digital health) and their application to produce and leverage healthcare data. In the context of LHS, the competency of informatics refers to learning how to use information systems to conduct research in LHS settings and to improve systems and patient results [5]. Effective training in informatics is an essential requirement for medical trainees and early career medical professionals to become LHS experts and for academic medical centers to become LHS [6].

### 1.1 Clinical Informatics Training in Medical Education

#### 1.1.1 Definition of Clinical Informatics

Clinical informatics (CI) is defined as “an interprofessional practice that blends medical practice with information technologies and behavioral management principles.” [7] By using this definition, we align our study with a focus on the value of information to expand medical research and improve healthcare delivery and patient outcomes, rather than focusing on a pre‐established set of technical tools or academic activities [7].

#### 1.1.2 CI Training for Medical Students

Informatics training can be difficult to access at the Undergraduate Medical Education (UME) level. This may be due to factors such as the constricted schedule of the medical school curriculum, technical difficulties, and lack of clinical faculty with sufficient health informatics knowledge to design and implement a curriculum [8]. A survey of four U.S. medical schools found increased interest from students in CI training, but also a lack of awareness of corresponding training opportunities [9]. Examples of existing UME training opportunities are CI special interest groups [10] and self‐paced online didactic courses [11].

Previous studies have indicated the need to provide medical students with more hands‐on experience with EHR systems [12] and practical skills in interpreting big datasets as a tool for clinical decision‐making [13, 14]. Other studies have highlighted how medical students make limited use of advanced features like clinical decision support systems (CDSS) [13, 15] and how informatics training should be incorporated as part of clinical rotations rather than stand‐alone courses [13, 16, 17]. These gaps are associated with clinician frustration in using information technology [12, 16, 18] and risks for patient safety [15, 16, 18].

#### 1.1.3 CI Training for Postgraduate Medical Professionals

Medical professionals from any specialty can pursue CI training at the Graduate Medical Education (GME) level, and CI was established as its own subspecialty by the American Board of Medical Specialties in 2010 [19, 20]. While post‐graduate medical professionals can pursue this specialty, many studies indicate a continued lack of effective training in CI for post‐graduate professionals. For example, a UK‐based study found health informatics competencies set by the International Medical Informatics Association were not sufficiently represented in clinical curricula for GME [21]. Another study highlighted the few opportunities for physician trainees to be exposed to clinical informatics [22].

### 1.2 Previous Literature Reviews

There have been various reviews (e.g., systematic, scoping, literature, and review of existing courses) addressing the topic of CI training in UME and GME [23, 24, 25, 26, 27, 28, 29, 30, 31, 32, 33]. Many of these have focused on electronic health records/electronic medical records as a CI tool, while others have focused on Artificial Intelligence (AI), telemedicine/telehealth, digital health, and clinical informatics tools in general (e.g., mobile health). To our knowledge, this is the first review that is not limited to a single CI tool *and* targets both UME and GME.

Among three reviews focusing on electronic health records (EHRs), there was little evidence to support the effectiveness of existing training programs [23, 24, 25]. Reviews focusing on AI as a CI tool have highlighted data analytics and visualization as critical skills for medical students [28]. Identified challenges to CI education included a need for faculty trained in CI and significant investment in technology tools [29]. Overall, despite advances in training, little is still known about the best practices for and the impact of informatics education for clinician skill development.

## 2 Question(s) of Interest

To inform the current gap in informatics skills training in the clinical setting, we aim to answer the following research questions: (1) How are academic medical institutions implementing CI education initiatives? (2) Are these initiatives implementing recommended LHS Informatics competencies? and (3) How effective are these initiatives according to established educational evaluation frameworks? Our objective is to summarize training program characteristics pertaining to CI and map these to the Kirkpatrick educational evaluation model and the AHRQ informatics competencies. This review can assist schools for health professions in teaching clinicians how best to leverage informatics tools and improve health systems at multiple levels, from individual patient care to system‐wide quality improvement projects. It can also provide health professions education researchers with a broad overview of current methods and tools used in education for this domain, promoting inter‐institutional and interprofessional collaboration.

## 3 Methods

Our literature search criteria in the simplest form focused on studies that: (a) included medical students and/or postgraduate medical professionals as part of the study sample, (b) took place in an academic medical setting, and (c) described a CI educational initiative.

To guide our research team in the screening process, we expanded our research objectives using simple definitions. This allowed our team to understand the *who*, which included postgraduate medical professionals and medical students, and our *what* to be defined as any study mentioning curriculum design and delivery for medical education on clinical informatics. In our search, we considered all types of tools and software (e.g., EHRs and AI) to reflect the wide array of informatics tools used in the clinical setting. For example, our review included articles showcasing AI applications relevant to CI such as Clinical Decision Support Systems and EHR data analytics [28]. We defined the *where* by limiting our search to curricula being implemented in academic settings, including both medical schools and academic medical centers. For the purposes of this review, we use the Institute of Medicine's definition of an academic medical center, which consists of a teaching hospital affiliated with a medical school [34]. We applied this definition to both national and international institutions. In preparation for screening, we also realized there were many types of educational approaches. We specified the type of studies we wanted to include by defining *how c*linical informatics curricula can be delivered, this included but was not limited to traditional didactic lectures, multimedia educational materials, online learning modules, interactive learning activities, fellowships, and bootcamps. We excluded studies that did not meet any of these criteria.

**FIGURE 1:**
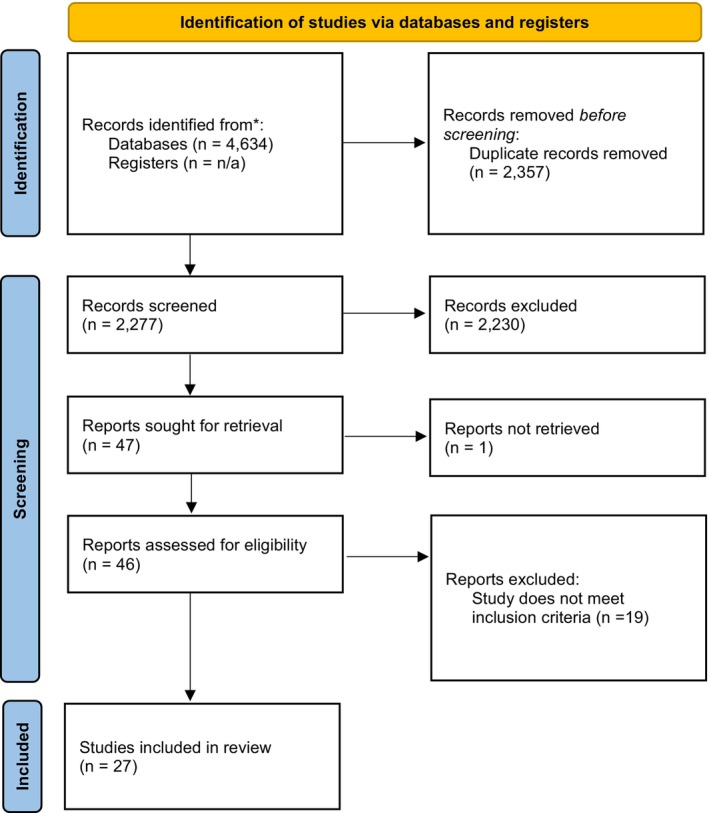
PRISMA flow diagram.

An information specialist from the Texas Medical Center Library (KLK) developed the search strategy for Embase (Appendix A in Supporting Information). Additionally, AZ searched the databases Ovid Medline and Web of Science (search strategies in Appendix B in Supporting Information). All searches to retrieve the citations were conducted between July 1 and July 7, 2024, after which we removed duplicates. Three researchers (AZ, LW, LT) independently screened titles and abstracts and selected articles for inclusion. Discrepancies were resolved through consensus. Once articles were selected for the second stage of screening, a full‐text review was done. Data were extracted by author AZ from articles meeting inclusion criteria, including study location, size of the CI training program, key informatics tools and methods used in the program, and aim of the curriculum. This was checked for internal consistency by having the second author (LT) extract data from 50% of the final set of articles. Curriculum aims were extracted from information in the text of the article, and then, as part of our data analysis, common aims were grouped together. We followed the Preferred Reporting Items for Systematic Review and Meta‐Analyses (PRISMA) flow diagram to summarize our search workflow (Figure 1).

During article screening and selection, we focused on articles published within the last 5 years to reflect the fast‐paced nature of CI tools. As an example, EHRs, one of the most prevalent types of CI tools, are constantly evolving to meet new clinical and patient needs and to support the business needs of healthcare organizations. This translates into an ongoing quest to produce tools with optimal user‐centered designs, features, and interfaces [35]. Therefore, curricular endeavors in CI need to adapt constantly and stay up to date regarding these tools.

We appraised curricula for effectiveness through Kirkpatrick's framework [36] and the Informatics competencies established by AHRQ for Learning Health Systems [5]. Kirkpatrick's framework evaluates training programs according to four levels: (1) Reaction: the extent to which participants found the training relevant, engaging, and favorable, (2) Learning: attaining skills, knowledge, attitude, confidence, and commitment because of the training, (3) Behavior: participants apply the obtained learning once out of the training, and (4) Results: the training results in organizational outcomes. Our curricula appraisal using Kirkpatrick's framework consisted of extracting available information from the article text describing an evaluation done, and then matching this evaluation and any corresponding results to the highest represented Kirkpatrick level. Table 1 provides a conceptual representation of this process. The first author (AZ) conducted the Kirkpatrick appraisal of all final selected articles, and of these, the second author (LT) appraised a subset of 50% of randomly selected articles to ensure internal consistency. Authors reached 100% agreement on their Kirkpatrick appraisal results.

**TABLE 1:** Curriculum appraisal using Kirpatrick's framework (examples).

Reference #	Evaluation reported in article	Evaluation results (if any)	Matching Kirkpatrick Level
[37]	Self‐reported student confidence in understanding AI and ML, initial feedback from students qualitatively through one‐on‐one meetings.	Significant increase in understanding of AI/ML from pre‐intervention (mean = 2.5 out of 5) to post‐intervention (mean = 4.1).	Level II: Learning
[38]	Pre‐post confidence surveys	Significant increase (p‐value ranges from 0.001 to 0.04) in resident's confidence in understanding AI concepts	Level II: Learning

The AHRQ Informatics Competency Domain contains five competencies focusing on the use of EHR data and other additional sources to conduct research and quality improvement, and to advance population health. The competencies also focus on knowledge of clinical information systems (e.g., EHRs, Computerized Physician Order Entry).

## 4 Results

Our literature search resulted in a total of 4634 articles, and after deduplication, there were 2277 articles to be screened (Figure 1). Using our inclusion criteria, we excluded 2230 articles during title and abstract screening. We sought to retrieve the full text from 47 articles, of which one was not immediately available for analysis. We analyzed the full text of 46 articles, of which 19 were excluded based on our selection criteria. We included a total of 27 articles for analysis (Table 2 of Appendix C in Supporting Information).

### 4.1 Study Characteristics

Of our selected studies, 18 were from North America [37, 38, 39, 40, 41, 42, 43, 44, 45, 46, 47, 48, 49, 50, 51, 52, 53, 54], eight from Europe [55, 56, 57, 58, 59, 60, 61, 62], and one from Asia [63]. Nine of the 27 studies focused on EHRs [41, 43, 44, 46, 47, 48, 51, 58, 59], seven on AI [37, 38, 39, 40, 50, 60, 62], three on the broad domain of digital health [57, 61, 63], three included multiple CI tools [49, 52, 55], two focused on telemedicine [42, 53], one on data science [56], one on clinical decision support [54], and one on the combination of clinical informatics training programs with various clinical fellowships [45].

### 4.2 Curriculum Characteristics

#### 4.2.1 Target Population

Curricula varied in terms of target population, including residents and fellows [38, 40, 42, 45, 46, 47, 51, 52], learners of all levels of UME [55, 59, 60, 63], and learners from specific years of UME [37, 39, 43, 44, 48, 50, 53, 54, 56, 58, 61, 62]. Only one curriculum targeted both UME and GME populations [41]. Two studies targeted students from various health professions including medicine [49, 57].

#### 4.2.2 Participants, Duration, and Requirement Status

The number of participants ranged from three psychiatry residents [52] to 2000 health professions students [57]. The length of curricula also varied greatly from a 75‐min workshop [44] to longitudinal 5‐year programs [55]. Most programs (*n* = 17) were elective [37, 38, 39, 40, 41, 42, 43, 44, 46, 47, 53, 54, 57, 59, 61, 62, 63], whereas 10 were required [45, 48, 49, 50, 51, 52, 55, 56, 58, 60].

#### 4.2.3 Objectives

There was a wide range in the breadth and depth of objectives implemented. For example, some curricula provided a survey‐level introduction to CI topics. Other curricula focused on very specific tools and technical skills, such as involving participants in AI‐based clinical decision support development. In Table 2, we illustrate our grouping of the curriculum objectives into four categories: Basic knowledge acquisition, Data analytics, Development of other technical skills, and Program Planning. Most curricula (*n* = 16) fell under the category of Basic knowledge acquisition.

**TABLE 2:** Grouping of curriculum objectives.

Curriculum objective	Curriculum objective examples
Basic knowledge acquisition [37, 38, 40, 41, 42, 45, 47, 48, 51, 52, 57, 58, 59, 60, 61]	To increase resident's foundational literacy on AI [38]. To provide an overview of Clinical Informatics and prepare those students interested in more detailed knowledge [41].
Data analytics [39, 55, 56, 63]	For students to learn how data are combined, assessed, interpreted, presented, and leveraged for AI use [39].
Development of other technical skills [43, 44, 46, 49, 53, 62]	To improve student's skills in order entry and other tasks using the Epic EHR [43].
Program planning [50]	To improve implementation of preclinical telemedicine training by systematically detailing the format, components, and integration of a telemedicine program into previously existing competencies [50].

#### 4.2.4 Delivery

Most studies (*n* = 17) used a combination of both didactic and practical mechanisms for curriculum delivery [37, 38, 39, 40, 41, 42, 44, 48, 50, 52, 53, 54, 55, 56, 57, 58, 62]. Practical aspects of delivery included group and lab meetings [37, 39, 48, 55, 59, 62] workshops [40, 49, 63], and informatics projects and exercises [37, 38, 42, 44, 45, 50, 51, 52, 58, 60]. One study mentioned how the COVID‐19 pandemic affected curriculum delivery from traditional classroom lectures to online formats of delivery including synchronous virtual meetings and asynchronous video lectures [55].

#### 4.2.5 Evaluation

Not all studies conducted an evaluation of their curriculum intervention, and those that did used different evaluation methods. For example, many curricula were evaluated through students' self‐reported confidence and self‐efficacy [37, 38, 42, 43, 49, 53, 54]. Only three studies incorporated more objective measures, such as knowledge assessments [39, 40, 42]. Many studies showed positive evaluation results, such as increased student confidence and knowledge of CI tools [37, 38, 39, 42, 43, 48, 49].

### 4.3 Analysis Using AHRQ Informatics Competencies

All but three of the curricula [37, 38, 57] incorporated the competency “Demonstrate knowledge of clinical information systems.” Sixteen curricula incorporated the competency “Demonstrate the ability to assess data quality and apply data quality assurance processes.^”^ [37, 39, 41, 42, 44, 46, 47, 48, 52, 54, 56, 59, 61, 62] Fourteen curricula incorporated the competency of using data derived from EHRs and other clinical information sources for research and quality improvement [37, 39, 41, 42, 44, 46, 47, 48, 52, 54, 56, 59, 61, 62]. Eight studies incorporated the competency of population health informatics (e.g., disease surveillance and monitoring) [39, 44, 46, 48, 55, 57, 61, 62]. Three studies had learners demonstrate knowledge about additional data sources to complement clinical data and enhance exposure and outcome assessment [55, 57, 62]. Figure 2 of Appendix D in Supporting Information illustrates the count of curricula that incorporated each AHRQ competency.

### 4.4 Analysis Using the Kirkpatrick Framework

The outcomes of most studies (*n* = 12) were related to the Kirkpatrick Level II of Learning [37, 40, 43, 44, 45, 47, 49, 54, 56, 57, 58, 59]. Ten studies reported outcomes related to Kirkpatrick Level III of Behavior [38, 39, 41, 42, 46, 48, 50, 52, 53, 62]. Four studies reported outcomes related to the Kirkpatrick Level I of Reaction [55, 60, 61, 63]. One study showed outcomes related to Level IV of Results [51]. Figure 3 of Appendix D in Supporting Information represents the distribution of studies among levels of the Kirkpatrick model.

## 5 Discussion

This review characterizes existing CI training in UME and GME and presents key topics to guide future education and research programs. The range of curriculum characteristics highlights the diverse nature of the field.

We found several gaps that should be addressed in future programs and research. The literature we reviewed is mostly focused on the Learning and Behavior levels of Kirkpatrick's model, while very few studies discussed how the training helped advance health outcomes at the organizational level (Kirkpatrick level of Results). Furthermore, few studies discussed plans to transform organizations into LHS through the implementation of informatics competencies or to complement existing clinical data with additional data sources to advance population health and disease monitoring efforts. However, we also noted a trend of CI tools being increasingly used in the context of research and applied projects such as the building of decision support systems [62].

Our findings parallel previous literature indicating evaluation methods for CI training could be more robust and should use more objective measures of evaluation in addition to participant self‐reported confidence [64]. Very few studies incorporated measures such as a comparison group or a technical assessment of participants' performance on tasks such as developing an algorithm or a clinical decision support tool. Therefore, the development of standardized evaluation measures to assess the efficiency of curricula for CI training at the UME and GME levels is needed. Some studies indicated certain CI tasks are already being evaluated as part of medical entrustable professional activities (EPAs), such as entering orders in the EHR [48].

### 5.1 Barriers to CI Education

Barriers to CI education among the studies selected include a lack of uniformity in the implementation of CI education. For example, medical schools can widely vary in their use of EHR software and the modalities through which they teach these skills to their students. There is also a need for more international and industry collaboration, as we observed that academic institutions are currently siloed in their efforts implementing CI education initiatives. Our selected articles also indicate CI education requires high levels of operational effort (i.e., coordination among IT staff and faculty and students). Other barriers encountered were: the complexity of CI tasks included in curricula (not being able to achieve CI learning objectives due to logistical, technical, or resource constraints), having no support from faculty (faculty's lack of interest or ability to develop and implement CI curricula), legal, ethical, and privacy issues (not being able to achieve CI learning objectives due to policies or regulations), passive learning (students losing interest in CI curriculum due to inactivity), and limited evidence supporting CI training (scarce literature or other academic work supporting CI training).

### 5.2 Facilitators to CI Education

One of the most important facilitators to CI education was enthusiasm and engagement from faculty and students. These provide momentum for establishing CI training programs and obtaining buy‐in from institutional leadership. As examples, two of our selected articles [48, 63] highlight the importance of commitment and enthusiasm from both faculty and students in CI programs to increase the program's success.

Other facilitators to CI education that we noted were self‐paced and flexible curricula, sharing of resources (such as having open‐source academic EHRs), and offering professional development opportunities (e.g., continuing medical education credits (CME), protected learning time, and interdisciplinary collaboration).

### 5.3 Recommendations for CI Education

Based on our identified barriers and facilitators to CI education, we ideated recommendations for future initiatives in this domain. These are outlined in Table 3. Our main recommendations include the following: (a) due to the fast‐paced evolution of CI tools, curricula should not focus entirely on technical programming but rather on critical thinking, thereby supporting long‐term clinician success, (b) while change to an already taut educational timeline and dense medical curriculum may seem difficult, planned CI curricula can flourish if incorporated into existing coursework without requiring much additional academic time and if practical learning activities are implemented multiple times throughout the academic year, and (c) we also recommend CI education transition from elective to required content for all medical students and residents to address the lack of uniformity in the implementation of CI education initiatives.

**TABLE 3:** Example recommendations to address barriers for CI education.

Barrier	Recommended solution	Example solution
Diverse and fast‐paced nature of CI technical tools	Emphasize activities that require critical thinking as part of technical exercises.	Include a post‐intervention knowledge quiz that asks: “In your own words, how would you address issues of data integrity and interoperability in your clinical practice?”
Limited time within the medical curriculum for informatics training	Incorporation of CI concepts into existing coursework	Students complete activity on ordering medications using EHR during Internal Medicine clinical rotations
Operational challenges	Initiatives to increase resources for CI education and research	A medical school starts a CI student coalition with the goal of facilitating fundraising activities and access to CI resources.
Need for more international and industry collaborations	Establishing research collaboration agreements across institutions and with industry	An EHR software company establishes an agreement with a medical school to sponsor training software tools for its students
Lack of uniformity in implementation of CI education	Establishing CI as required educational component of medical curricula, and collaboration between regulating organizations to produce standardized assessments	Inclusion of CI questions as part of USMLE examinations

Addressing these barriers to CI training is important because previous research has indicated that clinicians who receive this training may have improved job satisfaction, a more efficient clinical workflow, and increased personal wellness [43].

## 6 Limitations

Our study is not without limitations. A risk for individual and error bias was introduced with the first author conducting the assessment methods for the complete final set of articles selected. We have addressed this limitation by having the second author do a secondary assessment of 50% of the set, greatly reducing this risk. An additional limitation is that we did not search gray literature, nor did we search references of selected articles for additional articles to include. However, this study does provide a broad overview of existing peer‐reviewed literature regarding CI curricula and how they fit within education evaluation and clinical informatics competency frameworks. Lastly, we want to note that our discussion themes of barriers, facilitators, and recommended solutions for CI education are based on the limited literature available on this nascent topic. For example, our recommended solution to focus on critical thinking is largely based on the Kirkpatrick score and results for one of our selected studies [51] rather than an aggregation of all studies due to their wide variability in study design. Such discussion themes can be further quantified and made more robust as the topic grows in the literature.

Future work in this domain may include additional data sources, including gray literature. Future literature reviews on this topic might build upon our work in searching based on a more specific selection such as a specific topic (e.g., EHR, AI education), or discipline (e.g., pharmacy, nursing, radiology, ultrasound).

## 7 Conclusion

This work assessed the current state of Clinical Informatics training programs for a Learning Health System. Gaps remain in the context of CI education initiatives to promote the transformation of academic medical centers into LHS, improve healthcare quality, and achieve organizational‐level results. To mitigate these gaps, we recommend future work consider barriers and facilitators to CI education identified in this review. Our study suggests CI educational initiatives for UME and GME would benefit from the use of more comprehensive evaluation methods and the standardization of educational content. This would elevate the rate of CI education implementation, while simultaneously advocating for UME and GME programs.

## Author Contributions

**Alexandra Zingg:** conceptualization, project administration, data curation, methodology, writing – original draft, formal analysis. **L. Ida Tovar:** writing – original draft, writing – review and editing, formal analysis, project administration. **Laura Witte:** formal analysis, writing – review and editing. **Kelsey L. Koym:** conceptualization, methodology, data curation, writing – review and editing, visualization. **Kyler Godwin:** conceptualization, project administration, methodology, writing – review and editing, formal analysis, supervision.

## Funding

This work was supported by the U.S. Department of Veterans Affairs (3HPECCD2020).

## Conflicts of Interest

The authors declare no conflicts of interest.

## Supporting information


**Appendix A:** Supporting Information.

**Appendix B:**Supporting Information.

**Appendix C:** Supporting Information.

**Appendix D:** Supporting Information.

## Data Availability

The data that supports the findings of this study is available in the Supporting Information of this article.
