# Supplementary material for: Clinical Informatics Education to Advance Learning Health Systems: A Scoping Review
Source: Learn Health Syst. 2025 Dec 5;10(1):e70050. doi: 10.1002/lrh2.70050 (PMC12812492; doi:10.1002/lrh2.70050)
Supplement: Supplementary file 1 — Appendix A: Supporting Information. [file LRH2-10-e70050-s003.docx]

**From:** [no_reply@embase.com](mailto:no_reply@embase.com)

**Sent:** Friday, July 5, 2024 3:31 PM

**To:** Zingg, Alexandra

**Subject:** Embase Search Queries - Search

*****CAUTION:*** This email is not from a BCM Source. Only click links or open attachments you know are safe.**

**
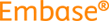
**

**Search Queries**

| **No.** | **Query** | **Results** | **Date** |
| --- | --- | --- | --- |
| #20 | (('medical informatics'/exp OR ('me | dical informatic*':ti,ab,kw 5289 | 5 Jul 2024 |

OR 'clinical informatic*':ti,ab,kw OR 'clinical information science*':ti,ab,kw OR 'clinical information tech*':ti,ab,kw OR 'health informatic*':ti,ab,kw OR 'health information science*':ti,ab,kw OR 'health information tech*':ti,ab,kw OR 'medical computer science*':ti,ab,kw OR 'medical data process*':ti,ab,kw OR 'medical informatics app*':ti,ab,kw OR 'medical informatics comput*':ti,ab,kw OR 'medical information science*':ti,ab,kw OR 'medical information tech*':ti,ab,kw OR 'public health informatic*':ti,ab,kw OR infodemiolog*:ti,ab,kw OR 'translational bioinformatic*':ti,ab,kw OR 'informatics education*':ti,ab,kw) OR 'biomedical informatic*':ti,ab,kw) AND ((('medical school'/exp OR 'medical education'/de OR 'clinical education'/exp OR 'clinical supervision'/de OR 'physician assistant education'/de OR 'residency education'/de OR 'teaching round'/de) OR ('medical school*':ti,ab,kw OR 'medical college*':ti,ab,kw OR 'medical universit*':ti,ab,kw OR 'medical research center*':ti,ab,kw OR 'medical academ*':ti,ab,kw OR 'medical curricul*':ti,ab,kw OR 'medical education*':ti,ab,kw OR 'clinical education*':ti,ab,kw OR 'academic medical institution*':ti,ab,kw)) OR ('curriculum'/de AND (('medical school'/exp OR 'medical education'/de OR 'clinical education'/exp OR 'clinical supervision'/de OR 'physician

assistant education'/de OR 'residency education'/de OR 'teaching round'/de) OR ('medical school*':ti,ab,kw OR 'medical college*':ti,ab,kw OR 'medical universit*':ti,ab,kw OR 'medical research center*':ti,ab,kw OR 'medical academ*':ti,ab,kw OR 'medical curricul*':ti,ab,kw OR 'medical education*':ti,ab,kw OR 'clinical education*':ti,ab,kw OR 'academic medical institution*':ti,ab,kw))) OR ('tertiary education'/de OR 'graduate education'/exp OR 'postdoctoral education'/exp OR 'postgraduate education'/exp) OR ('medical personnel'/de OR 'medical assistant'/de OR 'medical expert'/de OR 'medical specialist'/de OR 'medical student'/exp OR 'physician'/exp OR 'physician assistant'/de OR 'resident'/de) OR ('medical profession*':ti,ab,kw OR 'medical student*':ti,ab,kw OR 'postgraduate medical professional*':ti,ab,kw OR 'medical residen*':ti,ab,kw OR (((medical OR clinical OR clinic) NEAR/3 (faculty OR staff OR student* OR administrator* OR adiminstration* OR resident* OR residenc*)):ti,ab,kw) OR (((postgraduat* OR 'post-graduat*' OR postdoctor* OR 'post-doctoral*' OR graduate OR undergrad* OR fellow* OR professional*) NEAR/3 (medicine OR medical OR 'academic medic*')):ti,ab,kw)))) OR ((('medical informatics'/exp OR ('medical informatic*':ti,ab,kw OR 'clinical informatic*':ti,ab,kw OR 'clinical information science*':ti,ab,kw OR 'clinical information tech*':ti,ab,kw OR 'health informatic*':ti,ab,kw OR 'health information science*':ti,ab,kw OR 'health information tech*':ti,ab,kw OR 'medical computer science*':ti,ab,kw OR 'medical data process*':ti,ab,kw OR 'medical informatics app*':ti,ab,kw OR 'medical informatics comput*':ti,ab,kw OR 'medical information science*':ti,ab,kw OR 'medical information tech*':ti,ab,kw OR 'public health informatic*':ti,ab,kw OR infodemiolog*:ti,ab,kw OR 'translational bioinformatic*':ti,ab,kw OR 'informatics education*':ti,ab,kw) OR 'biomedical informatic*':ti,ab,kw) AND ((('medical

school'/exp OR 'medical education'/de OR 'clinical education'/exp OR 'clinical supervision'/de OR 'physician assistant education'/de OR 'residency education'/de OR 'teaching round'/de) OR ('medical school*':ti,ab,kw OR 'medical college*':ti,ab,kw OR 'medical universit*':ti,ab,kw OR 'medical research center*':ti,ab,kw OR 'medical academ*':ti,ab,kw OR 'medical curricul*':ti,ab,kw OR 'medical education*':ti,ab,kw OR 'clinical education*':ti,ab,kw OR 'academic medical institution*':ti,ab,kw)) OR ('curriculum'/de AND (('medical school'/exp OR 'medical education'/de OR 'clinical education'/exp OR 'clinical supervision'/de OR 'physician assistant education'/de OR 'residency education'/de OR 'teaching round'/de) OR ('medical school*':ti,ab,kw OR 'medical college*':ti,ab,kw OR 'medical universit*':ti,ab,kw OR 'medical research center*':ti,ab,kw OR 'medical academ*':ti,ab,kw OR 'medical curricul*':ti,ab,kw OR 'medical education*':ti,ab,kw OR 'clinical education*':ti,ab,kw OR 'academic medical institution*':ti,ab,kw))) OR ('tertiary education'/de OR 'graduate education'/exp OR 'postdoctoral education'/exp OR 'postgraduate education'/exp) OR ('medical personnel'/de OR 'medical assistant'/de OR 'medical expert'/de OR 'medical specialist'/de OR 'medical student'/exp OR 'physician'/exp OR 'physician assistant'/de OR 'resident'/de) OR ('medical profession*':ti,ab,kw OR 'medical student*':ti,ab,kw OR 'postgraduate medical professional*':ti,ab,kw OR 'medical residen*':ti,ab,kw OR (((medical OR clinical OR clinic) NEAR/3 (faculty OR staff OR student* OR administrator* OR adiminstration* OR resident* OR residenc*)):ti,ab,kw) OR (((postgraduat* OR 'post-graduat*' OR postdoctor* OR 'post-doctoral*' OR graduate OR undergrad* OR fellow* OR professional*) NEAR/3 (medicine OR medical OR 'academic medic*')):ti,ab,kw)))) AND ('electronic health record'/syn OR ('electronic medical record*':ti,ab,kw OR 'electronic health

record*':ti,ab,kw OR ehr:ti,ab,kw OR ehrs:ti,ab,kw OR emr:ti,ab,kw OR emrs:ti,ab,kw OR 'digital health record*':ti,ab,kw OR 'digital medical record*':ti,ab,kw)))

| #19 | (('medical informatics'/exp OR ('medical informatic*':ti,ab,kw OR 'clinical informatic*':ti,ab,kw OR 'clinical information science*':ti,ab,kw OR 'clinical information tech*':ti,ab,kw OR 'health informatic*':ti,ab,kw OR 'health information science*':ti,ab,kw OR 'health information tech*':ti,ab,kw OR 'medical computer science*':ti,ab,kw OR 'medical data process*':ti,ab,kw OR 'medical informatics app*':ti,ab,kw OR 'medical informatics comput*':ti,ab,kw OR 'medical information science*':ti,ab,kw OR 'medical information tech*':ti,ab,kw OR 'public health informatic*':ti,ab,kw OR infodemiolog*:ti,ab,kw OR 'translational bioinformatic*':ti,ab,kw OR 'informatics education*':ti,ab,kw) OR 'biomedical informatic*':ti,ab,kw) AND ((('medical school'/exp OR 'medical education'/de OR 'clinical education'/exp OR 'clinical supervision'/de OR 'physician assistant education'/de OR 'residency education'/de OR 'teaching round'/de) OR ('medical school*':ti,ab,kw OR 'medical college*':ti,ab,kw OR 'medical universit*':ti,ab,kw OR 'medical research center*':ti,ab,kw OR 'medical academ*':ti,ab,kw OR 'medical curricul*':ti,ab,kw OR 'medical education*':ti,ab,kw OR 'clinical education*':ti,ab,kw OR 'academic medical institution*':ti,ab,kw)) OR ('curriculum'/de AND (('medical school'/exp OR 'medical education'/de OR 'clinical education'/exp OR 'clinical supervision'/de OR 'physician assistant education'/de OR 'residency education'/de OR 'teaching round'/de) OR ('medical school*':ti,ab,kw OR 'medical college*':ti,ab,kw OR 'medical universit*':ti,ab,kw OR 'medical research center*':ti,ab,kw OR 'medical academ*':ti,ab,kw OR 'medical curricul*':ti,ab,kw OR 'medical education*':ti,ab,kw OR 'clinical  education*':ti,ab,kw OR 'academic medical | 1007 | 5 Jul 2024 |
| --- | --- | --- | --- |

institution*':ti,ab,kw))) OR ('tertiary education'/de OR 'graduate education'/exp OR 'postdoctoral education'/exp OR 'postgraduate education'/exp) OR ('medical personnel'/de OR 'medical assistant'/de OR 'medical expert'/de OR 'medical specialist'/de OR 'medical student'/exp OR 'physician'/exp OR 'physician assistant'/de OR 'resident'/de) OR ('medical profession*':ti,ab,kw OR 'medical student*':ti,ab,kw OR 'postgraduate medical professional*':ti,ab,kw OR 'medical residen*':ti,ab,kw OR (((medical OR clinical OR clinic) NEAR/3 (faculty OR staff OR student* OR administrator* OR adiminstration* OR resident* OR residenc*)):ti,ab,kw) OR (((postgraduat* OR 'post-graduat*' OR postdoctor* OR 'post-doctoral*' OR graduate OR undergrad* OR fellow* OR professional*) NEAR/3 (medicine OR medical OR 'academic medic*')):ti,ab,kw)))) AND ('electronic health record'/syn OR ('electronic medical record*':ti,ab,kw OR 'electronic health record*':ti,ab,kw OR ehr:ti,ab,kw OR ehrs:ti,ab,kw OR emr:ti,ab,kw OR emrs:ti,ab,kw OR 'digital health record*':ti,ab,kw OR 'digital medical record*':ti,ab,kw))

| #18 | 'electronic health record'/syn OR ('electronic medical record*':ti,ab,kw OR 'electronic health record*':ti,ab,kw OR ehr:ti,ab,kw OR ehrs:ti,ab,kw OR emr:ti,ab,kw OR emrs:ti,ab,kw OR 'digital health record*':ti,ab,kw OR 'digital  medical record*':ti,ab,kw) | 142039 | 5 Jul 2024 |
| --- | --- | --- | --- |
| #17 | 'electronic medical record*':ti,ab,kw OR 'electronic health record*':ti,ab,kw OR ehr:ti,ab,kw OR ehrs:ti,ab,kw OR emr:ti,ab,kw OR emrs:ti,ab,kw OR 'digital health  record*':ti,ab,kw OR 'digital medical record*':ti,ab,kw | 129924 | 5 Jul 2024 |
| #16 | 'electronic health record'/syn | 64126 | 5 Jul 2024 |
| #15 | ('medical informatics'/exp OR ('medical informatic*':ti,ab,kw | 5272 | 5 Jul 2024 |

OR 'clinical informatic*':ti,ab,kw OR 'clinical information science*':ti,ab,kw OR 'clinical information tech*':ti,ab,kw OR 'health informatic*':ti,ab,kw OR 'health information science*':ti,ab,kw OR 'health information tech*':ti,ab,kw OR

'medical computer science*':ti,ab,kw OR 'medical data process*':ti,ab,kw OR 'medical informatics app*':ti,ab,kw OR 'medical informatics comput*':ti,ab,kw OR 'medical information science*':ti,ab,kw OR 'medical information tech*':ti,ab,kw OR 'public health informatic*':ti,ab,kw OR infodemiolog*:ti,ab,kw OR 'translational bioinformatic*':ti,ab,kw OR 'informatics education*':ti,ab,kw) OR 'biomedical informatic*':ti,ab,kw) AND ((('medical school'/exp OR 'medical education'/de OR 'clinical education'/exp OR 'clinical supervision'/de OR 'physician assistant education'/de OR 'residency education'/de OR 'teaching round'/de) OR ('medical school*':ti,ab,kw OR 'medical college*':ti,ab,kw OR 'medical universit*':ti,ab,kw OR 'medical research center*':ti,ab,kw OR 'medical academ*':ti,ab,kw OR 'medical curricul*':ti,ab,kw OR 'medical education*':ti,ab,kw OR 'clinical education*':ti,ab,kw OR 'academic medical institution*':ti,ab,kw)) OR ('curriculum'/de AND (('medical school'/exp OR 'medical education'/de OR 'clinical education'/exp OR 'clinical supervision'/de OR 'physician assistant education'/de OR 'residency education'/de OR 'teaching round'/de) OR ('medical school*':ti,ab,kw OR 'medical college*':ti,ab,kw OR 'medical universit*':ti,ab,kw OR 'medical research center*':ti,ab,kw OR 'medical academ*':ti,ab,kw OR 'medical curricul*':ti,ab,kw OR 'medical education*':ti,ab,kw OR 'clinical education*':ti,ab,kw OR 'academic medical institution*':ti,ab,kw))) OR ('tertiary education'/de OR 'graduate education'/exp OR 'postdoctoral education'/exp OR 'postgraduate education'/exp) OR ('medical personnel'/de OR 'medical assistant'/de OR 'medical expert'/de OR 'medical specialist'/de OR 'medical student'/exp OR 'physician'/exp OR 'physician assistant'/de OR 'resident'/de) OR ('medical profession*':ti,ab,kw OR 'medical student*':ti,ab,kw OR 'postgraduate medical professional*':ti,ab,kw OR 'medical residen*':ti,ab,kw OR

(((medical OR clinical OR clinic) NEAR/3 (faculty OR staff OR student* OR administrator* OR adiminstration* OR resident* OR residenc*)):ti,ab,kw) OR (((postgraduat* OR 'post-graduat*' OR postdoctor* OR 'post-doctoral*' OR graduate OR undergrad* OR fellow* OR professional*) NEAR/3 (medicine OR medical OR 'academic medic*')):ti,ab,kw)))

| #14 | (('medical school'/exp OR 'medical education'/de OR 'clinical education'/exp OR 'clinical supervision'/de OR 'physician assistant education'/de OR 'residency education'/de OR 'teaching round'/de) OR ('medical school*':ti,ab,kw OR 'medical college*':ti,ab,kw OR 'medical universit*':ti,ab,kw OR 'medical research center*':ti,ab,kw OR 'medical academ*':ti,ab,kw OR 'medical curricul*':ti,ab,kw OR 'medical education*':ti,ab,kw OR 'clinical education*':ti,ab,kw OR 'academic medical institution*':ti,ab,kw)) OR ('curriculum'/de AND (('medical school'/exp OR 'medical education'/de OR 'clinical education'/exp OR 'clinical supervision'/de OR 'physician assistant education'/de OR 'residency education'/de OR 'teaching round'/de) OR ('medical school*':ti,ab,kw OR 'medical college*':ti,ab,kw OR 'medical universit*':ti,ab,kw OR 'medical research center*':ti,ab,kw OR 'medical academ*':ti,ab,kw OR 'medical curricul*':ti,ab,kw OR 'medical education*':ti,ab,kw OR 'clinical education*':ti,ab,kw OR 'academic medical  institution*':ti,ab,kw))) | 478794 | 5 Jul 2024 |
| --- | --- | --- | --- |
| #13 | (('medical school'/exp OR 'medical education'/de OR | 1709423 | 5 Jul 2024 |

'clinical education'/exp OR 'clinical supervision'/de OR 'physician assistant education'/de OR 'residency education'/de OR 'teaching round'/de) OR ('medical school*':ti,ab,kw OR 'medical college*':ti,ab,kw OR 'medical universit*':ti,ab,kw OR 'medical research center*':ti,ab,kw OR 'medical academ*':ti,ab,kw OR 'medical curricul*':ti,ab,kw OR 'medical education*':ti,ab,kw OR

|  | 'clinical education*':ti,ab,kw OR 'academic medical institution*':ti,ab,kw)) OR ('curriculum'/de AND (('medical school'/exp OR 'medical education'/de OR 'clinical education'/exp OR 'clinical supervision'/de OR 'physician assistant education'/de OR 'residency education'/de OR 'teaching round'/de) OR ('medical school*':ti,ab,kw OR 'medical college*':ti,ab,kw OR 'medical universit*':ti,ab,kw OR 'medical research center*':ti,ab,kw OR 'medical academ*':ti,ab,kw OR 'medical curricul*':ti,ab,kw OR 'medical education*':ti,ab,kw OR 'clinical education*':ti,ab,kw OR 'academic medical institution*':ti,ab,kw))) OR ('tertiary education'/de OR 'graduate education'/exp OR 'postdoctoral education'/exp OR 'postgraduate education'/exp) OR ('medical personnel'/de OR 'medical assistant'/de OR 'medical expert'/de OR 'medical specialist'/de OR 'medical student'/exp OR 'physician'/exp OR 'physician assistant'/de OR 'resident'/de) OR ('medical profession*':ti,ab,kw OR 'medical student*':ti,ab,kw OR 'postgraduate medical professional*':ti,ab,kw OR 'medical residen*':ti,ab,kw OR (((medical OR clinical OR clinic) NEAR/3 (faculty OR staff OR student* OR administrator* OR adiminstration* OR resident* OR residenc*)):ti,ab,kw) OR (((postgraduat* OR 'post-graduat*' OR postdoctor* OR 'post-doctoral*' OR graduate OR undergrad* OR fellow* OR professional*) NEAR/3 (medicine OR medical OR 'academic  medic*')):ti,ab,kw)) |  |  |
| --- | --- | --- | --- |
| #12 | 'medical profession*':ti,ab,kw OR 'medical student*':ti,ab,kw OR 'postgraduate medical professional*':ti,ab,kw OR 'medical residen*':ti,ab,kw OR (((medical OR clinical OR clinic) NEAR/3 (faculty OR staff OR student* OR administrator* OR adiminstration* OR resident* OR residenc*)):ti,ab,kw) OR (((postgraduat* OR 'post-graduat*'  OR postdoctor* OR 'post-doctoral*' OR graduate OR | 222128 | 5 Jul 2024 |

undergrad* OR fellow* OR professional*) NEAR/3 (medicine OR medical OR 'academic medic*')):ti,ab,kw)

| #11 | 'medical personnel'/de OR 'medical assistant'/de OR 'medical expert'/de OR 'medical specialist'/de OR 'medical student'/exp OR 'physician'/exp OR 'physician assistant'/de  OR 'resident'/de | 1280960 | 5 Jul 2024 |
| --- | --- | --- | --- |
| #10 | 'tertiary education'/de OR 'graduate education'/exp OR 'postdoctoral education'/exp OR 'postgraduate  education'/exp | 25890 | 5 Jul 2024 |
| #9 | 'curriculum'/de AND (('medical school'/exp OR 'medical education'/de OR 'clinical education'/exp OR 'clinical supervision'/de OR 'physician assistant education'/de OR 'residency education'/de OR 'teaching round'/de) OR ('medical school*':ti,ab,kw OR 'medical college*':ti,ab,kw OR 'medical universit*':ti,ab,kw OR 'medical research center*':ti,ab,kw OR 'medical academ*':ti,ab,kw OR 'medical curricul*':ti,ab,kw OR 'medical education*':ti,ab,kw OR 'clinical education*':ti,ab,kw OR 'academic medical  institution*':ti,ab,kw)) | 47415 | 5 Jul 2024 |
| #8 | ('medical school'/exp OR 'medical education'/de OR 'clinical education'/exp OR 'clinical supervision'/de OR 'physician assistant education'/de OR 'residency education'/de OR 'teaching round'/de) OR ('medical school*':ti,ab,kw OR 'medical college*':ti,ab,kw OR 'medical universit*':ti,ab,kw OR 'medical research center*':ti,ab,kw OR 'medical academ*':ti,ab,kw OR 'medical curricul*':ti,ab,kw OR 'medical education*':ti,ab,kw OR 'clinical education*':ti,ab,kw OR 'academic medical  institution*':ti,ab,kw) | 478794 | 5 Jul 2024 |
| #7 | 'medical school*':ti,ab,kw OR 'medical college*':ti,ab,kw OR 'medical universit*':ti,ab,kw OR 'medical research center*':ti,ab,kw OR 'medical academ*':ti,ab,kw OR 'medical curricul*':ti,ab,kw OR 'medical education*':ti,ab,kw OR 'clinical education*':ti,ab,kw OR 'academic medical  institution*':ti,ab,kw | 217081 | 5 Jul 2024 |

| #6 | 'curriculum'/de | 115620 | 5 Jul 2024 |
| --- | --- | --- | --- |
| #5 | 'medical school'/exp OR 'medical education'/de OR 'clinical education'/exp OR 'clinical supervision'/de OR 'physician assistant education'/de OR 'residency education'/de OR  'teaching round'/de | 370747 | 5 Jul 2024 |
| #4 | 'medical informatics'/exp OR ('medical informatic*':ti,ab,kw OR 'clinical informatic*':ti,ab,kw OR 'clinical information science*':ti,ab,kw OR 'clinical information tech*':ti,ab,kw OR 'health informatic*':ti,ab,kw OR 'health information science*':ti,ab,kw OR 'health information tech*':ti,ab,kw OR 'medical computer science*':ti,ab,kw OR 'medical data process*':ti,ab,kw OR 'medical informatics app*':ti,ab,kw OR 'medical informatics comput*':ti,ab,kw OR 'medical information science*':ti,ab,kw OR 'medical information tech*':ti,ab,kw OR 'public health informatic*':ti,ab,kw OR infodemiolog*:ti,ab,kw OR 'translational bioinformatic*':ti,ab,kw OR 'informatics education*':ti,ab,kw)  OR 'biomedical informatic*':ti,ab,kw | 33511 | 5 Jul 2024 |
| #3 | 'biomedical informatic*':ti,ab,kw | 1153 | 5 Jul 2024 |
| #2 | 'medical informatic*':ti,ab,kw OR 'clinical informatic*':ti,ab,kw OR 'clinical information science*':ti,ab,kw OR 'clinical information tech*':ti,ab,kw OR 'health informatic*':ti,ab,kw OR 'health information science*':ti,ab,kw OR 'health information tech*':ti,ab,kw OR 'medical computer science*':ti,ab,kw OR 'medical data process*':ti,ab,kw OR 'medical informatics app*':ti,ab,kw OR 'medical informatics comput*':ti,ab,kw OR 'medical information science*':ti,ab,kw OR 'medical information tech*':ti,ab,kw OR 'public health informatic*':ti,ab,kw OR infodemiolog*:ti,ab,kw OR 'translational  bioinformatic*':ti,ab,kw OR 'informatics education*':ti,ab,kw | 15858 | 5 Jul 2024 |
| #1 | 'medical informatics'/exp | 24185 | 5 Jul 2024 |

For further information on email alerts please [visit the Embase Info site](https://urldefense.proofpoint.com/v2/url?u=https-3A__www.embase.com_info&d=DwMFAw&c=ZQs-KZ8oxEw0p81sqgiaRA&r=SAahFOcCUO-_4nO0Nliv1oapxHpsFON1xlawP1JHX2I&m=HZFXqADOe5WU-Aw_u2xKXJayZj3Yy8Suv_cJx7mvd3J9MgcsotX0PvKQo9mcV-LM&s=bpnwo-IJkys0CRHCC5zT6p8htDe9F3nZ5PoHsgz281Y&e).

[Embase](https://urldefense.proofpoint.com/v2/url?u=https-3A__www.embase.com_&d=DwMFAw&c=ZQs-KZ8oxEw0p81sqgiaRA&r=SAahFOcCUO-_4nO0Nliv1oapxHpsFON1xlawP1JHX2I&m=HZFXqADOe5WU-Aw_u2xKXJayZj3Yy8Suv_cJx7mvd3J9MgcsotX0PvKQo9mcV-LM&s=SbYxjwoLW89so8cueAajpmniBllDehdmXEFObA22V6U&e) provides access to more than 28 million validated biomedical and pharmacological records from Embase and MEDLINE.

This email has been sent to you via Embase, a product of Elsevier Life Sciences IP Limited.

You are receiving this email as a subscriber to Embase alerts. To unsubscribe, please follow the link at the top of this page.

Embase respects your privacy and does not disclose, rent or sell your personal information to any non-affiliated third parties without your consent, except as may be stated in the Embase [Privacy Policy](https://urldefense.proofpoint.com/v2/url?u=https-3A__www.elsevier.com_legal_privacy-2Dpolicy&d=DwMFAw&c=ZQs-KZ8oxEw0p81sqgiaRA&r=SAahFOcCUO-_4nO0Nliv1oapxHpsFON1xlawP1JHX2I&m=HZFXqADOe5WU-Aw_u2xKXJayZj3Yy8Suv_cJx7mvd3J9MgcsotX0PvKQo9mcV-LM&s=8aA7sF_0gGERYnym_bua8Yr8oKokPhk2IlNfHHO-A2I&e).

By using email or alert services, you agree to comply with the Embase [Terms and Conditions](https://urldefense.proofpoint.com/v2/url?u=https-3A__www.elsevier.com_legal_elsevier-2Dwebsite-2Dterms-2Dand-2Dconditions&d=DwMFAw&c=ZQs-KZ8oxEw0p81sqgiaRA&r=SAahFOcCUO-_4nO0Nliv1oapxHpsFON1xlawP1JHX2I&m=HZFXqADOe5WU-Aw_u2xKXJayZj3Yy8Suv_cJx7mvd3J9MgcsotX0PvKQo9mcV-LM&s=V25fTJHZdndE9fwQEP8ockKNybaXzFDhqEOCl64CO08&e).

Copyright © 2024 Elsevier Life Sciences IP Limited except certain content provided by third parties. Embase is a trade mark of Elsevier Life Sciences IP Limited.
