# Supplementary material for: Clinical Informatics Education to Advance Learning Health Systems: A Scoping Review
Source: Learn Health Syst. 2025 Dec 5;10(1):e70050. doi: 10.1002/lrh2.70050 (PMC12812492; doi:10.1002/lrh2.70050)
Supplement: Supplementary file 2 — Appendix B:Supporting Information. [file LRH2-10-e70050-s001.docx]

Table 1- Medline OVID Search Strategy

| **MEDLINE OVID** | | |
| --- | --- | --- |
|  | **Date: 07/05/24** |  |
| 1 | Medical Informatics/ | 13,129 |
| 2 | clinical informatic*.mp. | 857 |
| 3 | clinical information science*.mp. | 5 |
| 4 | clinical information tech*.mp. | 79 |
| 5 | health informatic*.mp. | 5,221 |
| 6 | health information science*.mp. | 27 |
| 7 | health information tech*.mp. | 4,464 |
| 8 | medical computer science*.mp. | 24 |
| 9 | medical data process*.mp. | 79 |
| 10 | medical informatics app*.mp. | 2,762 |
| 11 | medical informatics comput*.mp. | 811 |
| 12 | medical information science*.mp. | 54 |
| 13 | medical information tech*.mp. | 72 |
| 14 | public health informatic*.mp. | 1,536 |
| 15 | infodemiolog*.mp. | 830 |
| 16 | translational bioinformatic*.mp. | 195 |
| 17 | informatics education*.mp. | 323 |
| 18 | biomedical informatic*.mp. | 1,008 |
| 19 | 1 or 2 or 3 or 4 or 5 or 6 or 7 or 8 or 9 or 10 or 11 or 12 or 13 or 14 or 15 or 16 or 17 or 18 | 25,562 |
| 20 | medical school.mp. | 31,215 |
| 21 | Education, Medical/ | 62,187 |
| 22 | clinical education.mp. | 3,856 |
| 23 | clinical supervision.mp. | 1,997 |
| 24 | physician assistant education.mp. | 177 |
| 25 | residency education.mp. | 1,136 |
| 26 | "teaching round".mp. | 15 |
| 27 | medical college.mp. | 17,106 |
| 28 | medical research center*.mp. | 359 |
| 29 | medical academ*.mp. | 2,635 |
| 30 | academic medical institution*.mp. | 383 |
| 31 | (curriculum and (medical school or medical education or clinical education or clinical supervision or physician assistant education or residency education or teaching round or (medical school* or medical college* or medical research center* or medical education* or clinical education* or academic medical institution*))).mp. [mp=title, book title, abstract, original title, name of substance word, subject heading word, floating sub-heading word, keyword heading word, organism supplementary concept word, protocol supplementary concept word, rare disease supplementary concept word, unique identifier, synonyms, population supplementary concept word, anatomy supplementary concept word] | 24,389 |
| 32 | (tertiary education or graduate education or postdoctoral education or postgraduate education).mp. [mp=title, book title, abstract, original title, name of substance word, subject heading word, floating sub-heading word, keyword heading word, organism supplementary concept word, protocol supplementary concept word, rare disease supplementary concept word, unique identifier, synonyms, population supplementary concept word, anatomy supplementary concept word] | 6,455 |
| 33 | (medical personnel or medical assistant or medical expert or medical specialist or medical student or physician or physician assistant or resident).mp. [mp=title, book title, abstract, original title, name of substance word, subject heading word, floating sub-heading word, keyword heading word, organism supplementary concept word, protocol supplementary concept word, rare disease supplementary concept word, unique identifier, synonyms, population supplementary concept word, anatomy supplementary concept word] | 402,477 |
| 34 | (medical profession* or medical student* or postgraduate medical professional* or medical residen* or ((medical or clinical or clinic) adj3 (faculty or staff or student* or administrator* or administration* or resident* or residenc*)) or ((postgraduat* or post-graduat* or postdoctor* or post-doctoral* or graduate or undergrad* or fellow* or professional*) adj3 (medicine or medical or academic medic*))).mp. [mp=title, book title, abstract, original title, name of substance word, subject heading word, floating sub-heading word, keyword heading word, organism supplementary concept word, protocol supplementary concept word, rare disease supplementary concept word, unique identifier, synonyms, population supplementary concept word, anatomy supplementary concept word] | 245,734 |
| 35 | (electronic health record or (electronic medical record* or electronic health record* or ehr or ehrs or emr or emrs or digital health record* or digital medical record*)).mp. [mp=title, book title, abstract, original title, name of substance word, subject heading word, floating sub-heading word, keyword heading word, organism supplementary concept word, protocol supplementary concept word, rare disease supplementary concept word, unique identifier, synonyms, population supplementary concept word, anatomy supplementary concept word] | 79,818 |
| 36 | 20 or 21 or 22 or 23 or 24 or 25 or 26 or 27 or 28 or 29 or 30 or 31 or 32 | 131,640 |
| 37 | 33 or 34 | 602,310 |
| 38 | 19 and 35 and 36 and 37 | 27 |
| 39 | 19 or 35 | 101,077 |
| 40 | 36 and 37 and 39 | 496 |

Table 2- Web of Science Search Strategy

| **WEB OF SCIENCE** | | |
| --- | --- | --- |
|  | **Date: 07/05/24** |  |
| 1 | TS=(biomedical informatic*) | 2529 |
| 2 | TS=(medical informatic* OR clinical informatic* OR clinical information science* OR clinical information tech* OR health informatic* OR health information science* OR health information tech* OR medical computer science* OR medical data process* OR medical informatics app* OR medical informatics comput* OR medical information science* OR medical information tech* OR public health informatic* OR infodemiolog* OR translational bioinformatic* OR informatics education*) | 263802 |
| 3 | TS=(medical informatics) | 10047 |
| 4 | #1 OR #2 OR #3 Timespan: 2015-01-01 to 2024-07-04 | 167427 |
| 5 | TS=(electronic medical record OR electronic health record* OR ehr OR ehrs OR emr OR emrs OR digital health record* OR digital medical record*) | 97474 |
| 6 | #4 OR #5 | 252470 |
| 7 | ALL=(biomedical informatic*) | 21563 |
| 8 | ALL=(medical informatic* OR clinical informatic* OR clinical information science* OR clinical information tech* OR health informatic* OR health information science* OR health information tech* OR medical computer science* OR medical data process* OR medical informatics app* OR medical informatics comput* OR medical information science* OR medical information tech* OR public health informatic* OR infodemiolog* OR translational bioinformatic* OR informatics education*) | 1189404 |
| 9 | medical informatics (All Fields) | 70603 |
| 10 | #7 OR #8 OR #9 | 1193452 |
| 11 | TS=(curriculum) | 213199 |
| 12 | TS=(medical school OR medical education OR clinical education OR clinical supervision OR physician assistant education OR residency education OR teaching round) | 321053 |
| 13 | TS=(medical school OR medical college* OR medical universit* OR medical research center* OR medical academ* OR medical curricul* OR medical education*OR clinical education* OR academic medical institution*) | 315001 |
| 14 | #12 OR #13 | 521370 |
| 15 | #11 AND #14 | 42858 |
| 16 | TS=(medical personnel OR medical assistant OR medical expert OR medical specialist OR medical student OR physician OR physician assistant OR resident) | 914959 |
| 17 | TS=(medical profession* OR medical student* OR postgraduate medical professional* OR medical residen* OR ((medical OR clinical OR clinic) NEAR3 (faculty OR staff OR student* OR administrator* OR administration* OR resident* OR residenc*)) OR ((postgraduat* OR post-graduat* OR postdoctor* OR post-doctoral* OR graduate OR undergrad* OR fellow* OR professional*) NEAR3 (medicine OR medical OR academic medic*))) | 248820 |
| 18 | TS=(tertiary education OR graduate education OR postdoctoral education OR postgraduate education) | 104609 |
| 19 | #16 OR #17 OR #18 | 1066927 |
| 20 | #19 AND #15 AND #6 Timespan: 2015-01-01 to 2024-07-05 | 1917 |
