# Supplementary material for: Clinical Informatics Education to Advance Learning Health Systems: A Scoping Review
Source: Learn Health Syst. 2025 Dec 5;10(1):e70050. doi: 10.1002/lrh2.70050 (PMC12812492; doi:10.1002/lrh2.70050)
Supplement: Supplementary file 3 — Appendix C: Supporting Information. [file LRH2-10-e70050-s004.docx]

Table 2- Study Characteristics

| **Ref.** | **Region** | **Clinical Informatics Tool** | **Target Population** | **Curriculum Objective** | **Instructional Design** | **Participants** | **Duration** | **Kirkpatrick Level** |
| --- | --- | --- | --- | --- | --- | --- | --- | --- |
| 37 | North America | Artificial Intelligence | Senior Medical Students | Empower students to learn about the impact of AI and ML in their chosen specialty, enable students to have informed conversations about these technologies, and to establish a baseline level of understanding for all students. | Self-guided and web-based course, weekly web-based laboratory meetings and projects. | 19 | One month | Level II |
| 38 | North America | Artificial Intelligence | Residents | To increase resident's foundational literacy on AI | Lectures, case studies, programming exercises. | 12 | Three weeks | Level III |
| 39 | North America | Artificial Intelligence | Senior Medical Students | For students to learn how data are combined, assessed, interpreted, presented, and leveraged for AI use. | Videos, synchronous online lectures, weekly small group activities | 20 | Four weeks | Level III |
| 40 | North America | Artificial Intelligence | Residents | Focused on foundational knowledge of algorithms in AI. | Two formats: lecture series and an intensive workshop . | Approximately 40 | Six hours for the workshop, and seven months for the lecture series. | Level II |
| 41 | North America | Electronic Health Record | Undergraduate and graduate medical students (third and fourth year medical students, housestaff) | To provide an overview of Clinical Informatics and prepare those students interested in more detailed knowledge. | Didactic sessions, interactive learning activities | 23 | Four weeks | Level III |
| 42 | North America | Electronic Health Record | Residents | To provide basic knowledge about CI, encourage longitudinal learning, and incentivize application of CI skills/tools in the real world. | Informatics projects and didactic sessions | 31 | Two weeks or four weeks options | Level III |
| 43 | North America | Electronic Health Record | Second or Third Year Students | To improve student's skills in order entry and other tasks using the Epic EHR | Training on EHR integrated into established Family and Community Medicine clerkship. | 49 | Four weeks | Level II |
| 44 | North America | Various | Health Professions Students | Curriculum covers various clinical informatics topics with a focus on using an EHR where users can play the role of various professionals. | Online modules and embedded exercises | 31 | At discretion of each individual health professions school | Level II |
| 45 | North America | Artificial Intelligence | Residents | To expose radiology residents to AI decision support systems and enable them to make recommendations | Integration of a radiology AI decision support tool into clinical workflow | 15 | Two years | Level II |
| 46 | North America | Electronic Health Record | Senior Residents | To prepare Emergency Medicine residents in topics of order generation, workflow, clinical decision support, and user interface. | Training provided through vendor Epic (Epic Builder training). Planned curriculum includes biweekly meetings, assigned readings, implementation of local QI project. | 6 | Two four-day visits | Level III |
| 47 | North America | Various | Residents | Residents learn fundamentals of CI through attending the AMIA 10 x 10 course lecture series. The CI residency track has four pillars: service, education, research, and quality improvement. | Didactic course (AMIA 10 x 10) | 3 | Four years of residency | Level II |
| 48 | North America | Clinical Decision Support | First Year Medical Students | For pre-clerkship students to learn essentials of clinical informatics and clinical decision support as part of their gastroenterology module. | Didactic session and small group activity, with pre-work and previous presentations | 326 | 2.5 hours | Level III |
| 49 | North America | Electronic Health Record | Fourth Year Medical Students | To improve the performance of students in the task of order entry | Workshop | 366 | 75 minutes | Level II |
| 50 | North America | Telemedicine | First Year Medical Students | To improve implementation of preclinical telemedicine training by systematically detailing the format, components, and integration of their telemedicine program into previously existing competencies. | Problem-based learning, instructional videos, patient experiences, mentorship components - via in-person and asynchronous instruction. | None reported | 18 months | Level III |
| 51 | North America | Electronic Health Record | Residents | Providing additional education on EHR for more efficient use and improve wellness. | Brief, weekly meetings and 1 one-hour individualized meeting of each resident with an EHR trainer. | 26 | 5 months | Level IV |
| 52 | North America | Clinical Informatics training combined into a clinical fellowship | Fellows | To encourage CI training among fellows and serve as a guide for future program directors in future petitions for integrated training. | Four-year fellowship with three designs | 3 | 4 years | Level III |
| 53 | North America | Electronic Health Record | Second Year Medical Students | To increase student confidence in EMR documentation and clinical skills, increase patient facetime for supervising physicians, and uphold current patient satisfaction. | In-person training, didactic | 36 | 1 academic year | Level III |
| 54 | North America | Telemedicine | Fourth Year Medical Students | To improve student utilization of telemedicine technologies and factors affecting the patient experience. | Online orientation, patient experiences | 64 | 4-weeks | Level II |
| 55 | Europe | Various | All Medical Students | For students to learn how to interpret clinical trials and epidemiological research studies, including topics of data science, digital health, and evidence-based medicine. | Didactic lectures, large group work, and self-paced learning events. Pivoted to online learning during the COVID-19 pandemic. | 2593 | Five years | Level I |
| 56 | Europe | Data Science | Second Year Medical Students | Provide students with foundations and data skills for the data-driven medical setting. | Flipped classroom with online lectures and practical tutorials and computer labs (pair programming) using R and R Studio. | 630 | Six weeks | Level II |
| 57 | Europe | Digital Health | Health Professions Students | To train health professions students (including medical students) in various aspects of digital health | Video lectures and practical exercises | 2000 | 30 hours | Level II |
| 58 | Europe | Electronic Health Record | Fifth Year Medical Students | Add to existing knowledge on best practices to teach use of Electronic Health Records to medical students. | Didactic lecture and practical EHR exercises | 104 | Two days | Level II |
| 59 | Europe | Electronic Health Record | All Medical Students | For medical students to harness information from the EHR and become familiar with key EHR functions before clinical rotations. | Group activities using academic EHR | 24 | Unspecifi-ed number of weeks | Level II |
| 60 | Europe | Artificial Intelligence | All Medical Students | To enhance student knowledge of AI through incorporating specific case studies into existing curriculum. | Case-based learning | None reported | None reported | Level I |
| 61 | Europe | Digital Health | Third Year Medical Students | To show a proof of concept supporting an elective module on digital health education. | None reported | 32 | Three weeks | Level I |
| 62 | Europe | Artificial Intelligence | Fourth Year Medical Students | For students to develop an ability for critically designing and implementing AI-based decision support | Videos, group exercices | 15 | One Year | Level III |
| 63 | Asia | Digital Health | All Medical Students | To improve students' knowledge on medical big data and promote digital health skills. | Workshop | 274 | Two hours | Level I |
