# Supplementary material for: Clinical Informatics Education to Advance Learning Health Systems: A Scoping Review
Source: Learn Health Syst. 2025 Dec 5;10(1):e70050. doi: 10.1002/lrh2.70050 (PMC12812492; doi:10.1002/lrh2.70050)
Supplement: Supplementary file 4 — Appendix D: Supporting Information. [file LRH2-10-e70050-s002.docx]

Figure 2. Count of curricula that incorporated the AHRQ Learning Health System Informatics Competencies.

Figure 3. Categorization of studies according to the Kirkpatrick Model
